# A Room Temperature VOCs Gas Sensor Based on a Layer by Layer Multi-Walled Carbon Nanotubes/Poly-ethylene Glycol Composite

**DOI:** 10.3390/s18093113

**Published:** 2018-09-15

**Authors:** Zitao Liu, Tuoyu Yang, Ying Dong, Xiaohao Wang

**Affiliations:** 1 Graduate School at Shenzhen, Tsinghua University, University Town of Shenzhen, Shenzhen 518055, China; lzt17@mails.tsinghua.edu.cn (Z.L.); yty15@mails.tsinghua.edu.cn (T.Y.); xhwang@tsinghua.edu.cn (X.W.); 2 Tsinghua-Berkeley Shenzhen Institute, Tsinghua University, University Town of Shenzhen, Shenzhen 518055, China

**Keywords:** VOC, gas sensor, PEG, MWCNTs, room temperature

## Abstract

Sensitive detection of volatile organic compounds (VOCs) is significant for environmental monitoring and medical applications. In this work, multi-walled carbon nanotubes (MWCNTs) and polyethylene glycol (PEG) that have good adsorption for VOCs, were sprayed layer by layer on an interdigitated electrode (IDE) to build a sensitive VOCs gas sensor. The relative resistance change (△R/R) when the sensor was exposed to VOCs was measured. The sensor showed high sensitivity to acetone, ethanol, isopropanol and isoprene with fast response (110 ± 5 s) and recovery (152 ± 5 s) at room temperature, and the lower detection limit (LDL) of the sensor reached 9 ppm. With the micro-fabricated IDE structure, the sensor can be easily built into an electric nose for VOC recognition and measurement.

## 1. Introduction

The detection of VOCs in the environment or in exhaled breath has received considerable attention and is becoming increasingly important in the development of point of care testing (POCT) devices and Internet of Things (IoT) nodes [1,2,3,4,5,6,7]. The development of versatile materials enables advances in gas sensors or electric nose detection. Unique properties, including high sensing performance sensing, portability, and low power consumption are desired in sensors material and devices design. Metal oxide-based sensing materials have been investigated for more than 60 years and some of them are used in commercial gas sensing products [8]. For example, ZnO, SnO_2_ and WO_3_ -based gas sensors can detect several kinds of VOCs (e.g., isoprene, ethanol and acetone) at ppb levels [9,10,11]. However, these sensors still exhibit limitations such as poor selectivity and high operating temperatures (typically within the 300 to 500 °C range). This is because they are based on chemical oxidizing or reducing reactions between oxygen ions and the analyte, while VOCs have similar reducing reaction energies [12], and the high temperature is a necessary condition for generating oxygen ions. Hence, these disadvantages increase the power consumption and decrease the portability.

Polymers represent a class of VOC-sensing materials that can work at room temperature (RT). They have good absorption ability toward VOC molecules because the porous and fibrous structure of polymers can provide a large specific surface area, which can provide more adsorption sites. It was reported that a polymer coated silicon micro-ring resonator device achieved successful detection of VOCs at levels as low as 100 ppb [13]. However, this kind of sensor requires a complicated system to operate it and is thus not suitable for compact devices or IoT nodes. A chemiresistor has the advantage of constituting a miniaturized sensing system, and conductive polymers, carbon black polymers or metal nanoparticle polymer composites have been applied as sensing materials for chemiresistor-based VOC sensors [14]. Carbon nanotubes (CNTs) are used because of their good conductivity and intrinsic gas sensing properties [15,16]. Polymer are chosen as VOC adsorbents to enhance the sensitivity of sensors. Nanotube−polymer composites are therefore interesting topics to explore because of their potential gas sensing performance.

In addition to the type of sensing material, the material assembly method also affects the sensing properties of sensors because it has a significant influence on the final topography of the sensing material. Various techniques such as spray-coating, drop-coating, spin-coating, dip-coating and thermal evaporation have been applied to add sensing materials. Spray-coating is easy to operate and it can provide an ultrathin layer. The composite film is prepared by alternatively spraying MWCNT and polymer layers, producing a composite-like layer at the interface between the separately deposited materials [17].

**Figure 1:**
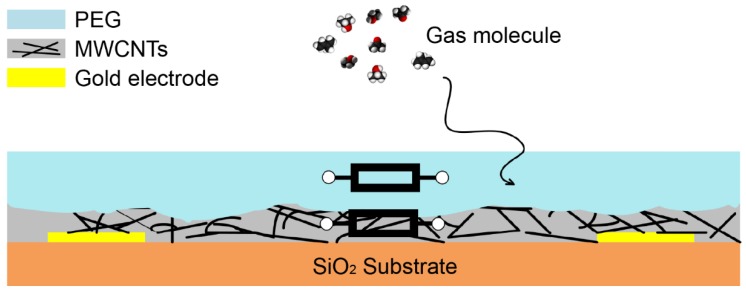
Schematic of the sprayed MWCNTs-PEG composite chemiresistive gas sensor.

In this work, a spraying process was applied to deposit two separate layers of MWCNT and polyethylene glycol (PEG) aiming to produce a composite-like structure that can act as sensing material for a chemiresistive sensor that is to be operated at room temperature. A schematic of the sensor is shown in Figure 1. Gas molecules can diffuse into the interior and are adsorbed on the surface of the sensing material where it reacts with the material. The response of the sensor when exposed to different concentrations of four VOCs (acetone, ethanol, isopropanol and isoprene) was investigated. Gas sensing characteristics such as the lower detection limit (LDL), response time, recovery time, reversibility features have been analyzed.

## 2. Materials and Methods

### 2.1. Materials and Characterization

Short multi-wall carbon nanotubes (MWCNTs), poly(acrylic acid) (PAA), polyethylenimine (PEI) and polyethylene glycol (PEG), acetone, ethanol, isopropanol, isoprene and sodium dodecyl sulfate (SDS) were purchased from Aladdin, Inc. (Shanghai, China) Morphological and structural characterization of the MWCNTs were carried out using Field Emission Scanning Electron Microscopy (SU8010, FESEM, Tokyo, Japan) and a BX53M Upright Metallurgical Microscope (Olympus, Tokyo, Japan). Molecular structure characterization of MWCNTs was carried out using Raman spectrometry (iHR320, HORIBA Scientific, Paris, France).

### 2.2. Gas Sensor Fabrication

**Figure 2:**
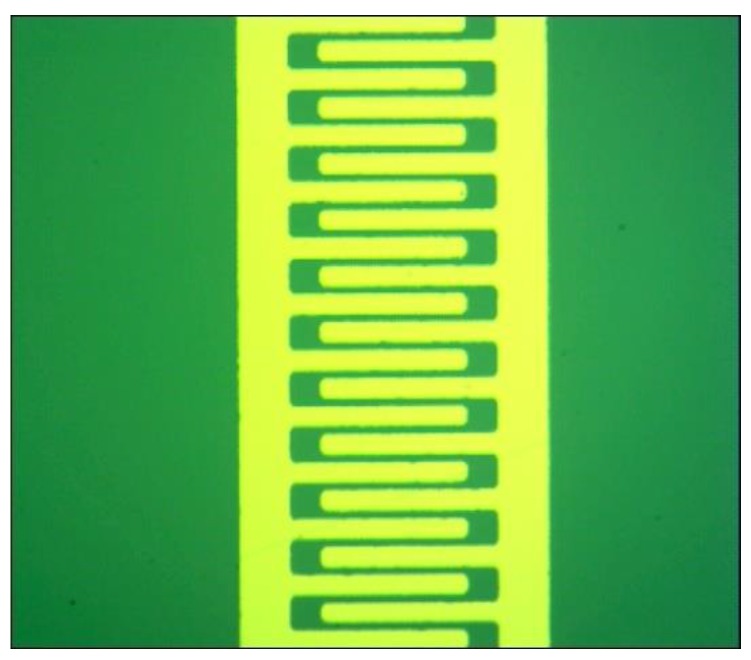
Optical micrograph of the IDE.

As shown in Figure 2, the interdigitated electrode (IDE) is 1 × 3 mm in size. Each finger gap is 25 μm. This type of geometry increases the contact area between the electrodes connected by the sensing film. The IDE was prepared on a silicon substrate with a 500 nm insulating SiO_2_ surface and it consisted of a 4 nm layer of Cr and a 100 nm layer of Au. The two metal layers were deposited on the surface by e-beam evaporation and patterned by photolithography and lift-off processes. The Cr layer was used to improve the adhesion of Au to the silicon dioxide layer. The Au layer was used as the contact layer to interface with the sensing material. The work function of Au matches the energy level of the MWCNTs so it tends to form an Ohmic contact between the electrode and the sensing material.

MWCNTs was dispersed in deionized water by ultrasonication with SDS (1:10) for 4 h. Then 3 wt % PEG was dissolved in deionized water. PAA and PEI are prepared in the same way as PEG. The IDEs were coated by spray-coating. The distance between the nozzle of the spray pen and substrate is 10 cm and the air pressure is 0.2 MPa. The coated area was defined by a mask and the layer thickness is controlled by the spray time, which in optimized experimental conditions is 2 s for each layer. Then the sensor was put on a hot plate at 55 °C for about 12 h to evaporate the solvent.

### 2.3. Gas Sensing Experiments

The sensor was placed in a test chamber (~50 mL volume) and its resistance was monitored continuously through a voltage divider method. The sensor was connected to a metal resistor whose resistance is about 10 times of the sensor’s resistance. The source was a NI PXI-4132 source unit (National Instruments, Austin, TX, USA) NI and the supply voltage is 5 V, and the voltage of the sensor was acquired. The signal was delivered to the digital interface (NI, PXIe-6368) connected to PC through a shielded cable. The data acquisition was performed by the user software programmed under LabVIEW (NI) environments with the sampling rate of 5 Hz. And the data were smoothed with their neighboring points by the Loess method to suppress rapidly-varying noise.

**Figure 3:**
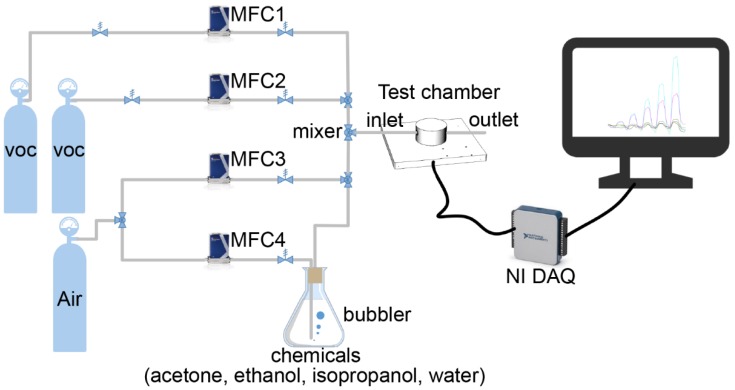
Schematic diagram of the experimental setup for gas sensing measurements.

To obtain the sensor response, the resistance was acquired as a reference baseline before the sensor was exposed to VOC gas. Then the sensor was cleaned with dry air for 15 min and after that it was tested upon exposure to alternating ca. 3 min cycles of VOC and dry air, respectively. The ethanol and acetone vapors were generated by passing carrier dry air through a bubbler which was placed in a 25 °C constant water bath and then diluted with carrier dry air. Each flow was controlled using programmable mass flow controller (MFC, Sevenstar Inc.,Beijing, China). Considering that the saturated vapor pressure of the VOC fluctuated with temperature or pressure fluctuations, a precise concentration of VOC was generated by dilution from the standard concentration VOC (100 ppm, HUATE GAS Inc., Shenzhen, China). The concentration of the diluted VOC was calculated by Equation (1):

(1) $$\;C_{diluted}=C_{0}\frac{F_{1}}{F_{1}+F_{2}}\;$$

where $F_{1}$ and $F_{2}$ are flow rate of VOC and carrier dry air representatively, and $C_{0}$ is 100 ppm here. The gas sensor testing platform is schematically represented in Figure 3. The sensor response of each test is calculated using Equation (2):

(2) $$\;S\left(\%\right)=\frac{R_{voc}-R_{0}}{R_{0}}\times 100\;$$

where $R_{voc}$ and $R_{0}$ are the resistance of the sensor in the presence of diluted VOC and dry air respectively. All the experiments have been carried out under room temperature and atmospheric conditions.

## 3. Results and Discussion

### 3.1. Device Fabrication

**Figure 4:**
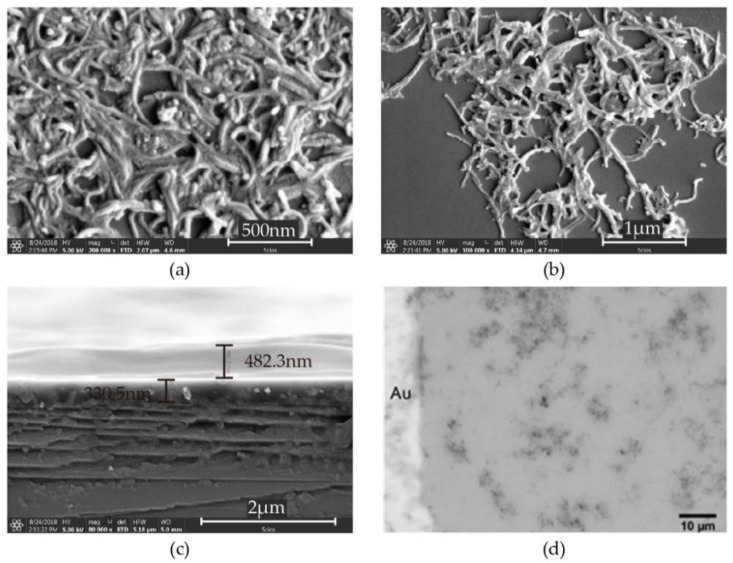
FESEM images of (**a**) MWCNTs on silicon dioxide; (**b**) PEG/MWCNTs on silicon dioxide; (**c**) the cross section of MWCNTs and PEG composite layer. (**d**) Optical microscope images of MWCNTs and PEG composite layer.

The FESEM image of the as prepared MWCNTs layer is shown in Figure 4a. The short and curved nanotubes formed a porous network. The MWCNTs seem like agglomerating. This may be due to the flow of the solvent evaporation. Figure 4b shows a FESEM image of the PEG/MWCNT composite layers. Spreading of PEG on the nanotubes network was observed. 

Figure 4c shows a cross-section of a MWCNTs and PEG composite layer. It was confirmed that the PEG/MWCNT composite consisted of a layer of MWCNTs and a layer of PEG, and MWCNT and PEG were mixed at the interface. The thickness of the MWCNTs layer was about 330.5 nm and the thickness of the PEG layer was approximately 482.3 nm. Figure 4d shows an optical microscopy image of the PEG/MWCNTs composite layers. From FESEM and the optical microscopy image it was confirmed that PEG was coated on the MWCNTs network. The morphology of the sensing material has an important role in determining a sensor’s performance. The crystalline structure and amorphous structure influence the VOC adsorption ability of material. With adsorbed VOC molecules, the nanostructure geometry and grain size vary and alter the electrical properties. The performances of the sensor, such as sensitivity, selectivity and response time, are directly related to the adsorption behaviors.

**Figure 5:**
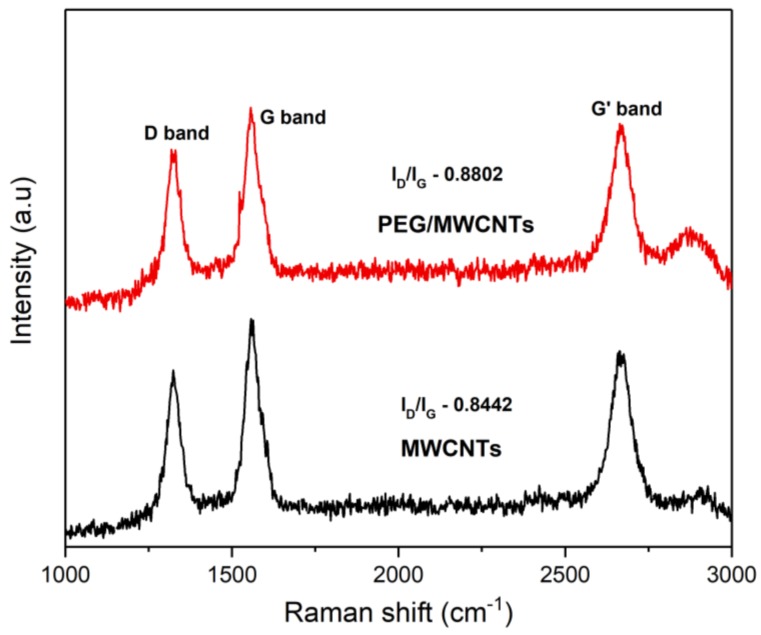
Raman spectra of MWCNTs and PEG/MWCNTs on silicon dioxide recorded from 1000 to 3000 cm^−1^ with calculated I_D_/I_G_ ratios.

Figure 5 shows the Raman spectra of the MWCNTs and PEG/MWCNTs on silicon dioxide. Prominent peaks were observed around 1350, 1580 and 2700 cm^−1^ corresponding to the D, G and G′ bands, respectively [18,19]. The intensity ratio of the D and G bands (I_D_/I_G_) from the Raman spectra of the MWCNTs and PEG/MWCNTs are 0.8442 and 0.8802, respectively. PEG/MWCNTs has a higher I_D_/I_G_ value, which indicates a PEG coating on the MWCNTs. A higher value of the I_D_/I_G_ is related to a high quantity of structural defects which provide more adsorption sites for VOCs.

The atomic composition of the short MWCNTs was examined by Energy Dispersive Spectroscopy (EDS). Samples were deposited onto silicon dioxide substrates. The results are summarised in Table 1. Short MWCNTs have been oxidized so they contain lots of oxygen species.

**Figure 6:**
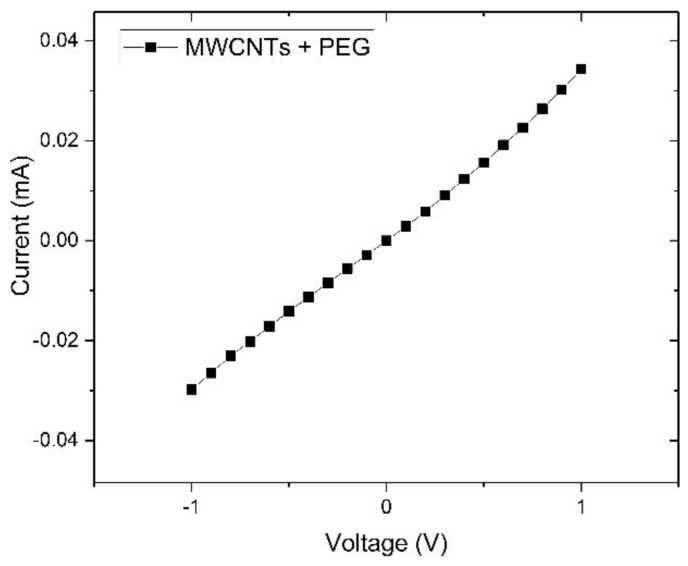
Current-voltage characteristic curve of PEG/MWCNTs.

Figure 6 shows linear increase in current-voltage characteristic of the composite film, which indicates it works as a resistor.

### 3.2. Sensor Response

**Figure 7:**
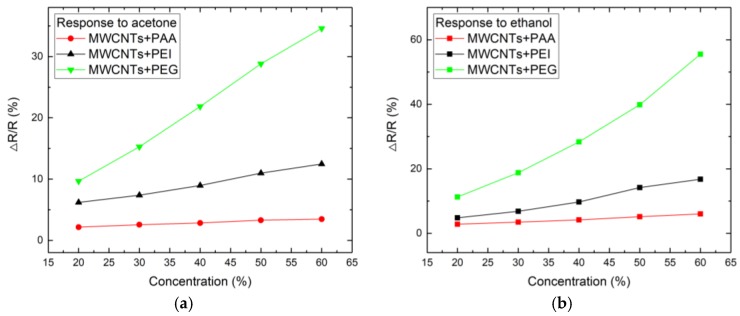
Sensor responses for various sensitive layer and vapors. The vapors corresponding to the figures are (**a**) acetone; (**b**) ethanol.

The responses of the sensors with different sensitive materials to different concentrations of acetone and ethanol are shown in Figure 7. The sensor based on MWCNTs and PEG composite has the highest sensitivity to the two vapors. The carboxyl group of the PAA polymer can combine with NH_3_ to form a new chemical bond. Amino functional groups of the PEI molecule can undergo a nucleophilic addition reaction. The PEG chain contains an oxygen atom to form a hydrogen bond [20]. These properties make the gas-sensitive properties of the four polymers different. The properties of PEG are more likely to produce higher sensitivity to VOCS.

**Figure 8:**
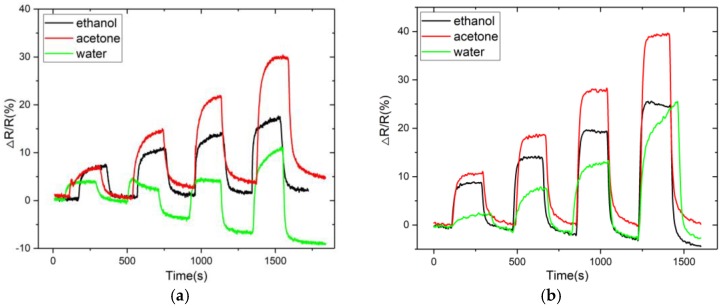
Sensor response comparison of (**a**) MWCNTs and (**b**) PEG/MWCNTs for various VOCs and water vapors.

**Figure 9:**
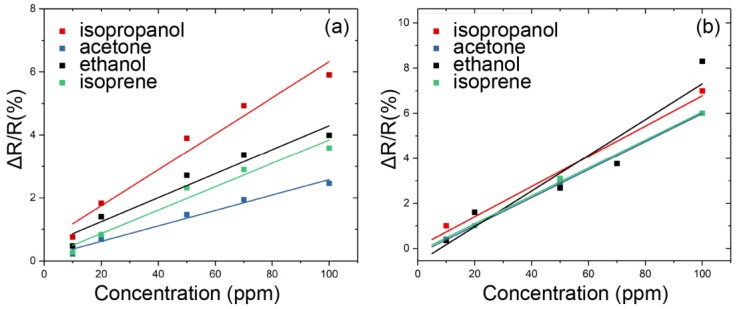
Fitting curves of the (**a**) MWCNTs and (**b**) PEG/MWCNTs sensor response as a function of VOCs concentration (10–100 ppm).

The sensor responses of the MWCNTs and PEG/MWCNTs upon exposure to 20% to 50% diluted VOC vapors and to water vapor at room temperature are shown in Figure 8a,b, respectively. It can be noticed from Figure 8 that PEG/MWCNTs on fabrics has higher response and smaller shift in the baseline when compared to naked MWCNTs. The sensor response to water vapor is obvious, implying that water vapor should be filtered out to obtain accurate VOC measurements. The responses of the MWCNTs and PEG/MWCNT sensors upon exposure to the four VOCs (isopropanol, acetone, ethanol and isoprene), at different concentrations (10, 20, 50, 70, 100 ppm) at room temperature are shown in Figure 9a,b, respectively. From Figure 9a, the linear correlation coefficient values of the fitting curves of isopropanol, acetone, ethanol and isoprene, are 0.965, 0.980, 0.949 and 0.967, respectively. From Figure 9b the linear correlation coefficient values are 0.981, 0.999, 0.913 and 0.997, respectively. This result indicates that sensor works linearly at low concentrations of isopropanol, acetone and isoprene, and it can be noticed that PEG/MWCNTs on fabrics has better linearity to isopropanol, acetone and isoprene when compared to naked MWCNTs. From Figure 9a, the slope values of the fitting curves of isopropanol, acetone and isoprene, are 0.0572, 0.0245 and 0.0372, respectively. The slope values are 0.0672, 0.0623 and 0.0620, respectively, in Figure 9b. These values represent the sensitivity of the sensor to VOCs. It is clear that PEG/MWCNTs sensor has better sensitivity when compared to naked MWCNTs.

According to the definition, the lower detection limit (LDL) was estimated by extrapolating the △R/R versus concentration curve to 3 σ/S (σ is the standard deviation of R, which was 0.178 here, and S is the slope of the fitting curve), and the LDL of the PEG/MWCNTs sensor to isopropanol, acetone and isoprene were 7.946, 8.571 and 8.613 ppm, respectively. The calculated LDL was not accurate enough as the gas line system was not perfectly accurate. But due to the high linear correlation coefficient values, the calculated LDLs make sense. These measurements as shown in Figure 8b present a shift in the baseline, which influences the estimation of the lower detection limit (LDL). This baseline shift is universally present for the gas sensor and the influence on the LDL is acceptable. The results implied that the composite provided large numbers of active sites to VOC molecules and the VOC molecules can diffuse into the composite, where they induced polymer swelling, which resulted in the conductive MWCNTs network being reconstructed and thus increasing the contact resistance of the MWCNT network.

**Figure 10:**
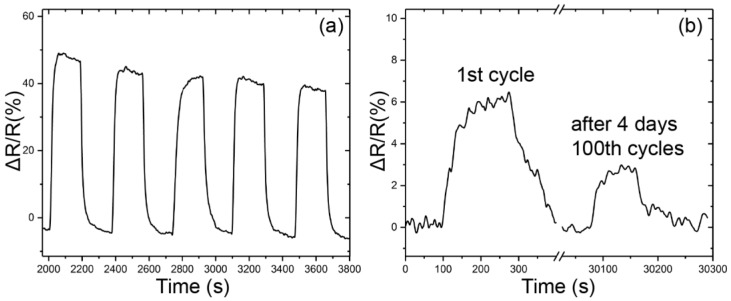
(**a**) Reversibility test for PEG/MWCNTs upon exposure to 50% diluted acetone; (**b**) Degradation of the sensor after 100 cycles upon exposure to 100 ppm of acetone.

Figure 10a shows the response recovery curve of the sensor exposed to 50% diluted acetone for five cycles. Although a baseline drift was observed, the reversibility of the response is acceptable. The response of the sensor upon exposure to low concentrations of VOCs is not stable at the beginning and then the response becomes stable. The response of the sensor to 100 ppm acetone dropped by half after 100 cycles, as shown in Figure 10b. The introduction of dry air allows the VOCs adsorbed on the sensitive material to be desorbed, thereby enabling reversible detection using the gas sensor. However, the effects of light, force, water vapor and heat may cause polymer aging, and in the process of detecting gases, resistance heat and swelling of VOCs and water vapor are inevitable. In a practical application, the sensor should work in a section with good long-term stability, and the device can be pre-aged during the production process.

The response and recovery time of the sensor are defined as the time taken for the sensor to reach the 90% saturation response value and the time taken for recovery to 10%, respectively. It was observed that the response/recovery time of the sensor was different when it was exposed to different concentrations of VOCs. It was about 60 ± 5 and 110 ± 5 s respectively for 50% diluted acetone vapor and this was increased to 110 ± 5 and 152 ± 5 s, respectively, for 100 ppm acetone. This may be attributed to the lower possibility of contact between the VOC molecules and sensing material at lower concentration.

**Figure 11:**
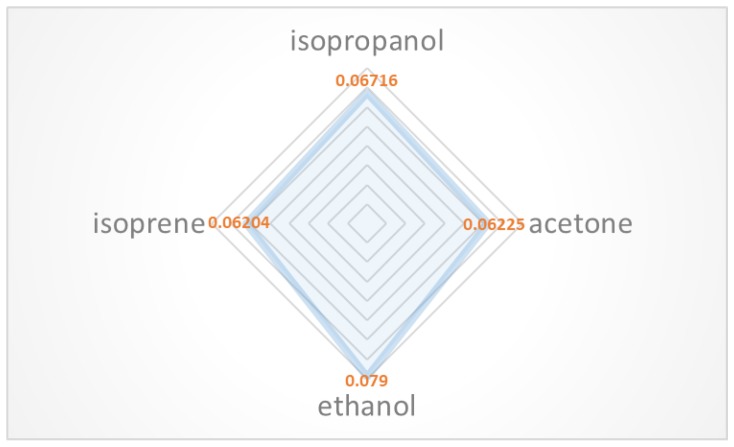
Radar plots of the response for four kinds of VOCs.

The normalized sensor response under exposure to 100 ppm of different gases is shown in Figure 11. This result indicates that the selectivity of the sensor is limited. This may be attributed to the similar properties of the tested VOCs and the fact their adsorption is universal. Compared to other MWCNT-based sensors or carbon material-based sensors reported in the literature, this work has achieved good LDL and sensitivity at room temperature. The comparison is shown in Table 2. Furthermore, due to the miniaturization of the sensor size and the simplicity of resistance measurement, this kind of sensor would be easily integrated into a sensor array and a micro-preconcentrator to achieve better discrimination and sensitivity [3,21,22].

## 4. Conclusions

A chemiresistive VOC sensor based on MWCNTs and PEG composite layers was designed and fabricated, and tested on different concentrations of four kinds of VOC (acetone, ethanol, isopropanol and isoprene). The sensor parameters such as LDL, reversibility, response and recovery time were analyzed. This sensor has shown good sensitivity and has the advantages of operating at room temperature, low power consumption and easy fabrication process. With the miniaturized sensor size and the simplicity of resistance measurement, this kind of sensor would be easy to integrate into a sensor array and it could be used for portable e-nose applications.

## Figures and Tables

**Table 1:** The atomic composition of the short MWCNTs.

Spectrum	Composition
C	32.14
O	67.86
Total	100

**Table 2:** Comparison of this work with various carbon nanotube-based gas sensors reported in the literature.

Sensing Materials	Operating Temp. (°C)	Concentration (ppm)	Sensitivity (ppm^−1^)	Resp/Recov. Time (s)	LDL (ppm)	Ref.
PSE/MWCNTs	RT	50–500	0.00007	--	50	[17]
Au/CNTs	RT	50–800	-	--	50	[23]
PVA/MWCNTs	RT	100–500	0.00005	26/30	100	[24]
ZnO/Graphene	280	10–10,000	0.123	2/1	<10	[25]
PEO/MWCNTs	RT	43–356	0.00022	275/300	43	[26]
IO-POSS/CNTs	RT	1.5–7	0.0025	--	1.5	[27]
PEG/MWCNTs	RT	10–1000	0.0006	110/152	<10	This work
